# Nucleases as a barrier to gene silencing in the cotton boll weevil, *Anthonomus grandis*

**DOI:** 10.1371/journal.pone.0189600

**Published:** 2017-12-20

**Authors:** Rayssa Almeida Garcia, Leonardo Lima Pepino Macedo, Danila Cabral do Nascimento, François-Xavier Gillet, Clidia Eduarda Moreira-Pinto, Muhammad Faheem, Angelina Maria Moreschi Basso, Maria Cristina Mattar Silva, Maria Fatima Grossi-de-Sa

**Affiliations:** 1 Brasilia Federal University (UnB), Brasília - CEP, Brasília, Federal District, Brazil; 2 Embrapa Genetic Resources and Biotechnology, Brasília, Federal District, Brazil; 3 Catholic University of Brasília, CEP, Brasília, Federal District, Brazil; University of Tennessee, UNITED STATES

## Abstract

RNA interference (RNAi) approaches have been applied as a biotechnological tool for controlling plant insect pests via selective gene down regulation. However, the inefficiency of RNAi mechanism in insects is associated with several barriers, including dsRNA delivery and uptake by the cell, dsRNA interaction with the cellular membrane receptor and dsRNA exposure to insect gut nucleases during feeding. The cotton boll weevil (*Anthonomus grandis*) is a coleopteran in which RNAi-mediated gene silencing does not function efficiently through dsRNA feeding, and the factors involved in the mechanism remain unknown. Herein, we identified three nucleases in the cotton boll weevil transcriptome denoted *AgraNuc1*, *AgraNuc2*, and *AgraNuc3*, and the influences of these nucleases on the gene silencing of *A*. *grandis* chitin synthase II (*AgraChSII*) were evaluated through oral dsRNA feeding trials. A phylogenetic analysis showed that all three nucleases share high similarity with the DNA/RNA non-specific endonuclease family of other insects. These nucleases were found to be mainly expressed in the posterior midgut region of the insect. Two days after nuclease RNAi-mediated gene silencing, dsRNA degradation by the gut juice was substantially reduced. Notably, after nucleases gene silencing, the orally delivered dsRNA against the *AgraChSII* gene resulted in improved gene silencing efficiency when compared to the control (non-silenced nucleases). The data presented here demonstrates that *A*. *grandis* midgut nucleases are effectively one of the main barriers to dsRNA delivery and emphasize the need to develop novel RNAi delivery strategies focusing on protecting the dsRNA from gut nucleases and enhancing its oral delivery and uptake to crop insect pests.

## Introduction

The availability of different insect’s transcriptomes allows the evaluation and identification of genes that can be potentially used for insect control using different biotechnological approaches [1–3]. To date, more than 100 insect transcriptomes have been deposited in databases, such as InsectBase [4], allowing the assessment of important and essential insect genes [3, 5–8].

The cotton boll weevil (CBW), *Anthonomus grandis*, belongs to the coleopteran order and is considered one of the most damaging insect pests to cotton crops, responsible for huge damages in cotton yields in South America [3], particularly in Brazil. Adult females lay eggs inside cotton buds and squares, where the larvae break out and feed on the plant tissue [9, 10]. This CBW’s endophytic habit substantially affects cotton buds flowering and thereby, the productivity, making its control difficult by using chemical pesticides. [11–13]. In optimal conditions, CBW takes approximately three weeks until reaching adulthood and each female can lay up to 300 eggs in cotton buds and squares, being able to generate 7 to 10 generations per year [3]. In normal conditions, the larva breaks out after three days of oviposition and immediately starts to feed. Approximately after 12 days, third instar larvae enter in pupa stage for five days and emerge as an adult. Adult CBW can live about 20 to 40 days, and its life cycle can last approximately 50 days [14].

In the past decade, Cry proteins from *Bacillus thuringiensis* (Bt) have been applied in the development of genetically modified (GM) cotton for CBW control [15–17]. An alternative approach for making GM cotton resistant to CBW is the application of RNA interference (RNAi) technology. RNAi is a promising alternative strategy for controlling crop insect pests that shows the advantage of using the insect’s systemic gene-silencing machinery to suppress essential gene expression [18–21]. Double-stranded RNA (dsRNA) is the RNAi trigger molecule that primes the post-transcriptional downregulation of a target gene [22–24]. Studies using this technology showed efficient silencing of several target genes through microinjection into larvae and adult CBW insects [1, 25, 26], whereas experiments involving the dsRNA oral uptake resulted in ineffective gene knockdown [18].

Efficient RNAi-induced gene silencing in insects requires some essential factors, including dsRNA processing by RNAi enzymes [27], intracellular transport [28], expression of the core RNAi machinery [29], delivery method [30], and uptake from the hemolymph or gut [31]. Different studies showed that the RNAi efficacy varies among insect species [32–38]. Wang and colleagues (2016) [39] confirmed this phenomenon in a parallel study with four different insect species from different orders: *Locusta migratoria* (Orthoptera), *Periplaneta americana* (Blattaria), *Spodoptera lituria* (Lepidoptera) and *Zophobas atratus* (Coleoptera). They demonstrated that the hemolymph and midgut juice content are key factors for RNAi efficacy. Therefore, dsRNA exposure should persist long enough to allow cellular uptake.

The uptake of dsRNA can be achieved by soaking, microinjection or feeding, and the RNAi signal is subsequently transported between cells and tissues [40]. In CBW, the dsRNA microinjection methodology is known to work well [1, 25, 26], while oral feeding is not so effective [18], and soaking methodology has not been tested. The oral ingestion of dsRNA is associated with some barriers, such as the exposure of the dsRNA to nucleases secreted in the gut juice, the gut pH, the dsRNA concentration and dsRNA resistance [38, 41, 42]. Furthermore, the gut juice content is also stimulated by increasing the nucleic acid ingestion through the diet [41, 43]. The nuclease activity of some insects was previously detected in the saliva of *Culex pipiens quinquefasciatus* [44], *Glossina morsitans* [45], and *Nezara viridula* [46]. Nucleases have also been identified in the *Bombyx mori* gastric juice [47] and the *Schistocerca gregaria* gut [38]. All of the identified nucleases belong to an ancient DNA/RNA sugar non-specific endonuclease family that is conserved in both prokaryotes and eukaryotes [48]. This family of proteins degrades dsDNA and dsRNA in a sequence-independent manner [49, 50]. Intriguingly, these nucleases have been identified in insects that evolved in phylogenetically distant orders, suggesting a potential conservation of these enzymes across insect taxonomy [38]. However, the identification and characterization of gut nucleases in insects are poorly studied.

To understand why the oral delivery of dsRNA has not succeeded in CBW, the presence of gut nucleases was evaluated. *In silico* approaches coupled with transcriptomic analyses allowed the identification of transcript sequences that encode putative extracellular DNA/RNA non-specific nucleases. Molecular and biochemical methodologies indicated the involvement of potential extracellular DNA/RNA non-specific nucleases in the degradation of dsRNA in the CBW gut. The data presented here indicate that the nucleases present in the CBW gut could be a major barrier to RNAi gene silencing in this insect pest.

## Materials and methods

### Rearing of cotton boll weevil

Cotton boll weevils were reared under controlled conditions of temperature (26 ± 2°C), relative humidity (70 ± 10%) and light (12 h photoperiod). They were fed daily with an artificial diet. The adults were developmentally synchronized by transferring them to a different cage after outbreak [51].

### Identification and in silico analysis of CBW nucleases

An extracellular dsRNase found in *B*. *mori* (NM_001098274.1) was used to identify orthologous proteins in the CBW transcriptome database [1] utilizing the tBLASTn tool (https://blast.ncbi.nlm.nih.gov/Blast.cgi). An open reading frame (ORF) cDNA search was performed using ORF Finder (https://www.ncbi.nlm.nih.gov/orffinder/). Nucleotide and peptide sequence alignments were executed with BioEdit (http://www.mbio.ncsu.edu/bioedit/bioedit.html) and the ClustalW algorithm (PAM250) [52]. Secondary structure prediction was performed on the GeneSilico protein structure Metaserver, which provides the mean secondary structure prediction from 16 different algorithms [53]. The signal peptide was predicted using the TargetP server [54]. Motifs were predicted with GLAM2 [55] using the 46 amino acid sequences. The phylogenetic analysis of the amino sequences from the nuclease catalytic domain was obtained using the phylogeny.fr server (one click mode) [56, 57]. The generated file was exported to the interactive Tree of Life (iTOL) software to view and format the constructed tree [58].

### RNA extraction and cDNA synthesis

Tissues of interest from CBW (anterior midgut, posterior midgut, posterior gut, whole gut and carcass) were dissected in 150 mM NaCl solution under an optical microscope and transferred to liquid nitrogen. RNA extraction from larvae and adult insects of CBW was performed with Trizol (Invitrogen), following the manufacturer’s recommended protocol. Equal quantities of RNA (2 μg) were used for cDNA synthesis with the M-MLV reverse transcriptase (Invitrogen) according to the manufacturer’s recommended protocol and 10 mM nvDT30 primer. The cDNA was stored at -20°C.

### Synthesis of dsRNA

Template DNA flanked by the T7 promoter sequence was synthesized to produce the dsRNA of CBW nucleases (*AgraNuc*1, *AgraNuc2* and *AgraNuc*3) and *chitin synthase II*, as well as *gus* dsRNA [26]. PCR reactions for the amplification of adult CBW cDNA were performed with specific primers (10 mM) for each gene (Table 1), an annealing temperature of 55°C, 10X buffer, 50 mM MgCl_2_, 10 mM dNTP and 1 U Taq polymerase (Invitrogen) following the manufacturer’s recommended protocol. The amplified products were analyzed by agarose gel electrophoresis (1%), and the amplified fragments were purified with a QIAquick PCR purification kit (Qiagen) and ligated into the PCR2.1 vector following the manufacturer’s recommended protocol (ThermoFisher). The ligation product was transformed into *Escherichia coli* XL1-Blue. The recombinant plasmid DNA was extracted from positive white colonies, and a PCR reaction was performed as described above with primers T7 and T7M13 Rv (Table 1). The amplified products were purified with a QIAquick PCR purification (Qiagen) kit, and the purified products were used as a template for dsRNA synthesis using the MEGAscript RNAi kit (Ambion), following the manufacturer’s recommended protocol. The dsRNAs length for AgraNuc1, AgraNuc2 and AgraNuc3 was, respectively, 291, 290 and 305 pb.

**Table 1:** Primer sequences.

Primer	Sequence (5' --> 3')
**AgraNuc1 Fw**	ATGTGACCACCGTAACCTGC
**AgraNuc1 Rv**	GTCTGTAGGAGGATTTGGAC
**AgraNuc2 Fw**	GACAATACGCTGCCAGAACTG
**AgraNuc2 Rv**	TCTATCGAAGGTGAGCCAGG
**AgraNuc3 Fw**	TTCTGAACGCGCTGCCTCAATG
**AgraNuc3 Rv**	GATCTGATTGGCTGTCTTG
**AgraChSII Fw**	AAGGCATTAACGGTGACGAC
**AgraChSII Rv**	TCCAAGTCGTTGATGACTGC
**nvDT30**	GAATTCACGCGTCGACTAGTAGCATATGTAC(T)30VN
**T7**	TAATACGACTCACTATAGGGAGA
**T7M13 Rv**	TAATACGACTCACTATAGGGAGACAGGAAACAGCTATGAC
**Agra-B-actin Fw**	GTAGCTCACGCCTCGGTACT
**Agra-B-actin Rv**	AGTGTTGGCCGAGGTATGAC
**Agra-β-tubulin Fw**	AGATCGTCGAGGGTCTGATG
**Agra-β-tubulin Rv**	AAGGCGGGAATGACTTTACC

### Extraction of midgut juice and nuclease assay

The gut juice from the adult CBW anterior midgut was extracted. The insects were anesthetized on ice for 10 minutes and dissected to collect the gut. The contents of the gut were extracted by grinding and resuspended in 150 mM NaCl solution. The total protein concentration was quantified using the Bradford method [59]. The nuclease assay was performed by incubating 500 ng of dsRNA or dsDNA with the gut juice sample (1 μg) in Triple Buffer, as described by [60], with modifications (10 mM sodium acetate, 10 mM Mes and 20 mM Tris), pH 5.5 at 37°C for 30 minutes in a reaction volume of 20 μL. To analyze its integrity, the nuclease digestion product was visualized in an agarose gel (1%). The optimum pH for CBW gut juice nuclease activity was determined by incubating 1 μg of dsRNA (~200 bp) with 2 μg of gut juice in Triple Buffer (pH was adjusted with HCl or NaOH, from 3.0 to 9.0). The reaction was incubated for 30 minutes in a reaction volume of 70 μL at 37°C. Then, dsRNA was purified with equal volume of phenol chloroform and chloroform and centrifuged for 10 minutes. Supernatant was removed and dsRNA was precipitated with equal volume of isopropanol and centrifuged for 10 minutes. Pellet was washed with ethanol 70% and centrifuged for 5 minutes. Supernatant was discarded and the pellet was diluted in milliQ water. Absorbance was plotted in a graph and the products were analyzed by agarose gel electrophoresis (1%). One activity unit (AU) of Nuclease is defined as the amount of enzyme that causes a ΔA260 of 0.1 in 30 min. The absorbance was measured at 260 nm in the NanoVue Plus spectrophotometer (GE Healthcare). All the assays were performed in triplicate, using 3.3 μg gut juice protein.

### Microinjection of dsRNA into the CBW body cavity

CBW young adults were anesthetized on ice for 10 minutes. Subsequently, 500 ng of dsRNA against each nuclease was administered through a 10 μL microinjection (syringe, Hamilton) into the insect body cavity, and these dsRNAs were injected both separately and in a mixture. The negative controls were microinjected with *gus* dsRNA. After the procedure, the insects were maintained under controlled conditions with respect to temperature (26 ± 2°C), relative humidity (70 ± 10%) and light (12-h photoperiod) for seven days.

### Analysis of nuclease expression patterns by quantitative real time-PCR

The relative gene expression of the nucleases in both CBW adults and larvae was validated by quantitative real-time PCR (RT-qPCR), and the relative expression levels were also measured in different sections of the gut (anterior midgut, posterior midgut and posterior gut), the whole gut and the carcass. Two and seven days after the microinjection, the insects were collected for gene silencing analysis. The RT-qPCR reactions were performed in a 7300 Real-Time PCR System (Applied Biosystems) using SYBR^™^ Green as the intercalating fluorophore. Specific primers for the nucleases (Table 1) were used. Each reaction was performed with 2 μL of diluted cDNA, 0.2 μM of each nucleotide and 2.5 μL of SYBR^™^ Green in a total volume of 10 μL.

The qRT-PCR program consisted of an initial step of 95°C for 10 minutes followed by 40 cycles of 95°C for 20 seconds, 55°C for 30 seconds and 72°C for 30 seconds. For the amplification analysis, the Ct value and amplification efficiency for each nucleotide (ranging from 90% to 100%) were determined using the Real-Time PCR Miner program (http://www.miner.ewindup.info/). The relative expression analysis based on the Ct values was performed with qBasePlus 2.0 using the Pfaffl method [61]. All qRT-PCR experiments included three biological replicates and three technical repetitions. The statistical analyses of the average nuclease expression levels were performed using Tukey’s test with a 0.05% significance level for comparisons between treatments. *Agra-β-actin* and *Agra-β-tubulin* were used as reference genes.

### Collection of the midgut juice two days after silencing and dsRNA digestion

Two days after the dsRNA injection, the intestinal juices of CBW adults were collected and incubated with dsRNA as described previously. All treatments were incubated for 30 minutes at 37°C with the gut juice from a total of 10 insects.

### *Chitin synthase II* transcription after *AgraNuc* dsRNA administration through feeding two days after nuclease gene silencing, mortality and oviposition

Two days after the microinjection of nuclease dsRNA into the CBW body cavity and verification of gene silencing, the insects were starved for two days. After this starvation period, 500 ng of dsRNA against *chitin synthase II (AgraChSII)* was administered in 5% sucrose. Three days after the oral administration of dsRNA, the whole insects were collected to evaluate the level of *AgraChSII* gene silencing through RT-qPCR. All qRT-PCR experiments included three biological replicates and three technical repetitions. Insects were counted every 24 hours to assess mortality and eggs were counted to evaluate oviposition.

## Results

### In silico analysis of nucleases found in CBW transcriptome shows a conserved endonuclease G domain and secretion signal peptides

**Fig 1:**
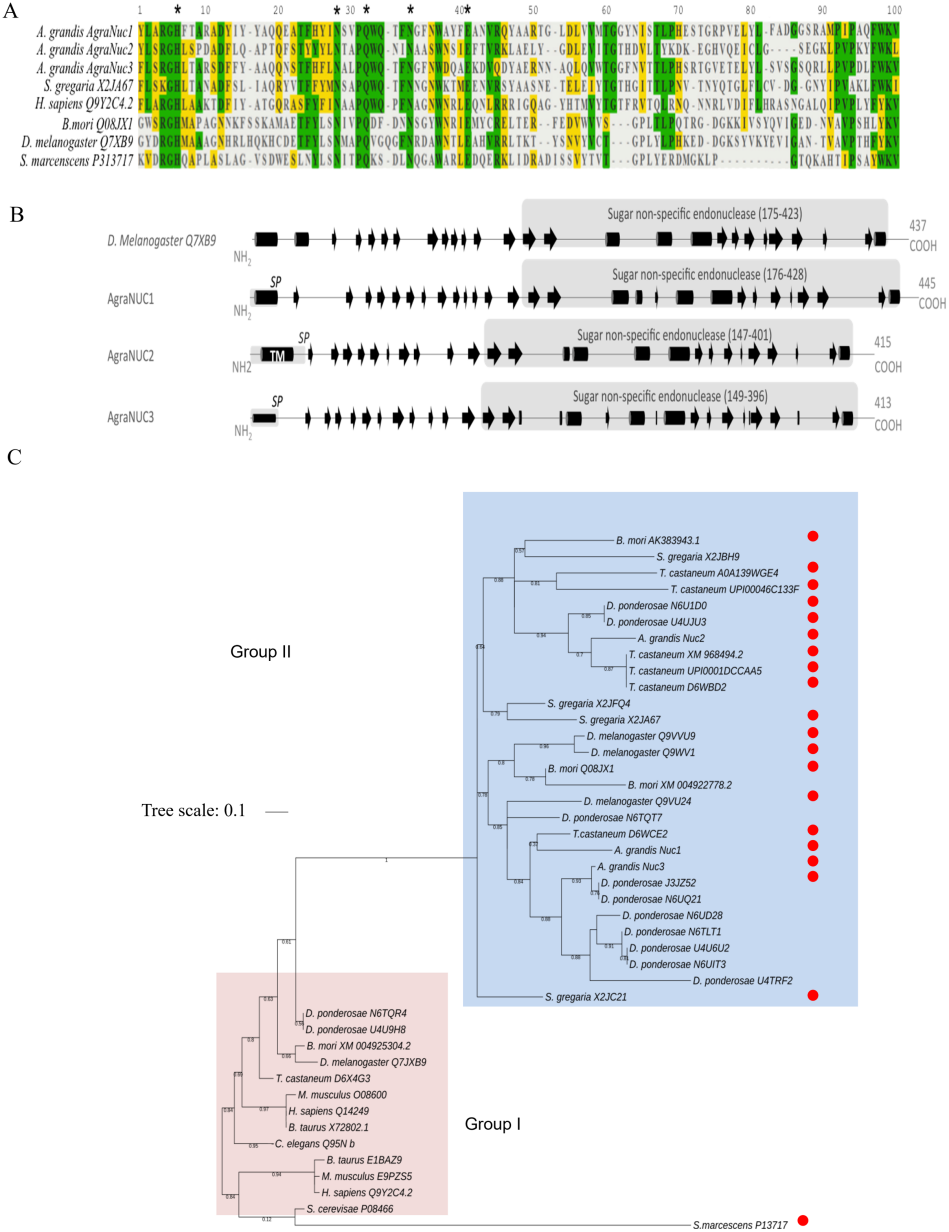
In silico analysis of candidate nucleases identified within the CBW gut. (A) Amino acid alignment of CBW nucleases with those from other insects and human sugar non-specific nucleases (*D*. *melanogaster*, *B*. *mori*, *S*. *gregaria*, *Mus musculus*, *B*. *taurus*, *H*. *sapiens*, and *S*. *cerevisiae*). The asterisks indicate the conserved motif Hx_22_Nx_4_Qx_5_Nx_8_E, which is involved in metal binding and catalysis. Identical and similar amino acids are highlighted in green and yellow, respectively. (B) CBW nucleases showing a C-terminus DNA/RNA sugar non-specific endonuclease domain and an N-terminus signal peptide. The black arrows and black cylinders represent the predicted β-strand and α-helix, respectively. “SP” is the predicted secretory peptide, and “TM” is a predicted transmembrane domain. The accession numbers refer to the UniProt protein data bank. (C) Maximum-likelihood phylogenetic tree of the non-specific endonuclease family divided by different animal species. Groups I and II are included in the red and blue areas, respectively. The red disks indicate the prediction of a secretory peptide (>0.7) at the N-terminal section of the nucleases. The accession numbers correspond to the UniProt or NCBI databases.

A tBLASTn analysis using a *B*. *mori* (NM_001098274.1) nuclease sequence as a template revealed three CBW ortholog genes named *AgraNuc1* (GABY01017164.1), *AgraNuc2* (GABY01017702.1) and *AgraNuc3* (GABY01006596.1). The molecular weight of the encoded proteins were approximately 45–50 kDa. Amino acid conservation is particularly relevant for the catalytic domain of proteins belonging to the sugar non-specific nuclease family [48]. Indeed, the motif Hx_22_Nx_4_Qx_5_Nx_8_E, where “x” represents any amino acid, is characteristic to this nuclease family and specifies the key residues directly involved in metal binding and catalysis [48, 60, 62, 63] (Fig 1A). Moreover, motif prediction showed that all three nucleases have a high probability of carrying a putative secretory signal peptide (SP) (cutoff >90) (Fig 1A). Secondary structure prediction showed similarities between these nucleases and the sugar non-specific endonuclease G (Endo G) from *Drosophila melanogaster* [62] (Fig 1B). Furthermore, signal peptide prediction indicated that *AgraNuc1-3* has a high probability of containing a putative secretory signal peptide (SP) (cutoff >0.7).

The sugar non-specific endonuclease family comprises different groups of nucleases with different specialized physiological functions such as cell death and nutrition [38, 62]. To study the phylogenetic relationships between the *AgraNuc1-3* genes and those from the non-specific endonuclease family, a multiple alignment analysis was performed using the amino acid sequences of the core catalytic domain (approximately 50 amino acids) of 44 nucleases that belong to different species (Fig 1C). Motif prediction of all the nucleases selected in this study confirmed the occurrence of the key residues from the core catalytic domain in this nuclease family (S1 Fig). The nucleases could be further divided into two main groups (I and II) with 50% bootstrap values. The observation of paralogs in different insect species, such as *Dendroctonus ponderosae* or *D*. *melanogaster*, suggests the occurrence of several duplication events in the evolutionary history of this gene family. Group I includes orthologs of the EndoG and ExoG proteins that were functionally characterized in *D*. *melanogaster* [62], *Saccharomyces cerevisiae* [64], *Caenorhabditis elegans* [65] and *Homo sapiens* [66]. These enzymes are reported to participate in cell death-related mechanisms [62]. Group II comprises *AgraNuc1-3* and other insect nucleases that have already been characterized as having a nutrition function in *S*. *gregaria* [38] and *B*. *mori* [47]. Similar to the findings for AgraNuc1-3, these two previous studies identified a secretory peptide at the N-terminal section of the protein. Interestingly, a secretory peptide has been predicted for a large number of nucleases that only belong to group II. Altogether, these data highlight the fact that *AgraNuc1-3* nucleases belong to a group of sugar non-specific nucleases that might have nuclease activity specifically in an extracellular environment.

### The gut juice from CBW presents nuclease activity

**Fig 2:**
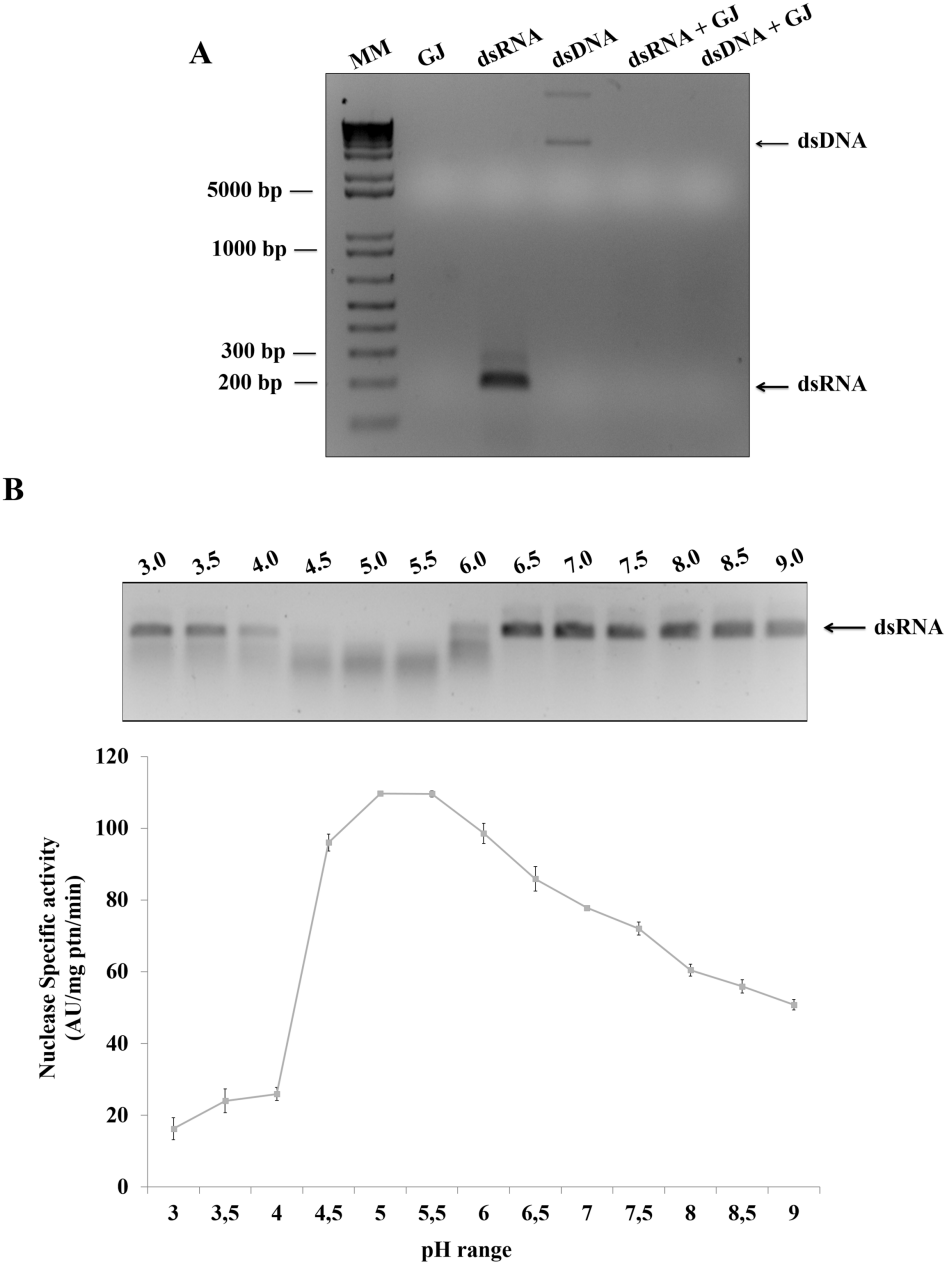
Biochemical characterization of CBW gut juice. (A) CBW gut juice (GJ), which is able to degrade both dsRNA, ~ 200bp, and dsDNA, > 5000 bp (as observed), has non-specific nuclease activity. MM: Molecular Marker 1-Kb Plus DNA ladder (Invitrogen); GJ: Gut Juice. Samples were incubated with GJ for 30 minutes at 37°C. (B) The optimal pH for nuclease activity ranges from 5.5 to 6.5, indicating that the nucleases function best at acidic pH.

In the attempt to identify the nuclease activity responsible for nucleic acid digestion in CBW, the gut juice was extracted from the insect anterior midgut (AMG) (S2 Fig). The ability of the CBW gut juice to digest different forms of nucleic acids was evaluated, and digestion was observed in a non-specific manner for both dsRNA and dsDNA (Fig 2A), which might demonstrate the presence of nucleases in the gut. Subsequently, the optimal pH for the gut juice nucleases was analyzed, and the results revealed that the nuclease activity is optimal in an acidic pH range from 5.5 to 6.5 (Fig 2B).

### *AgraNuc2* and *AgraNuc3* are highly expressed in the CBW posterior midgut

**Fig 3:**
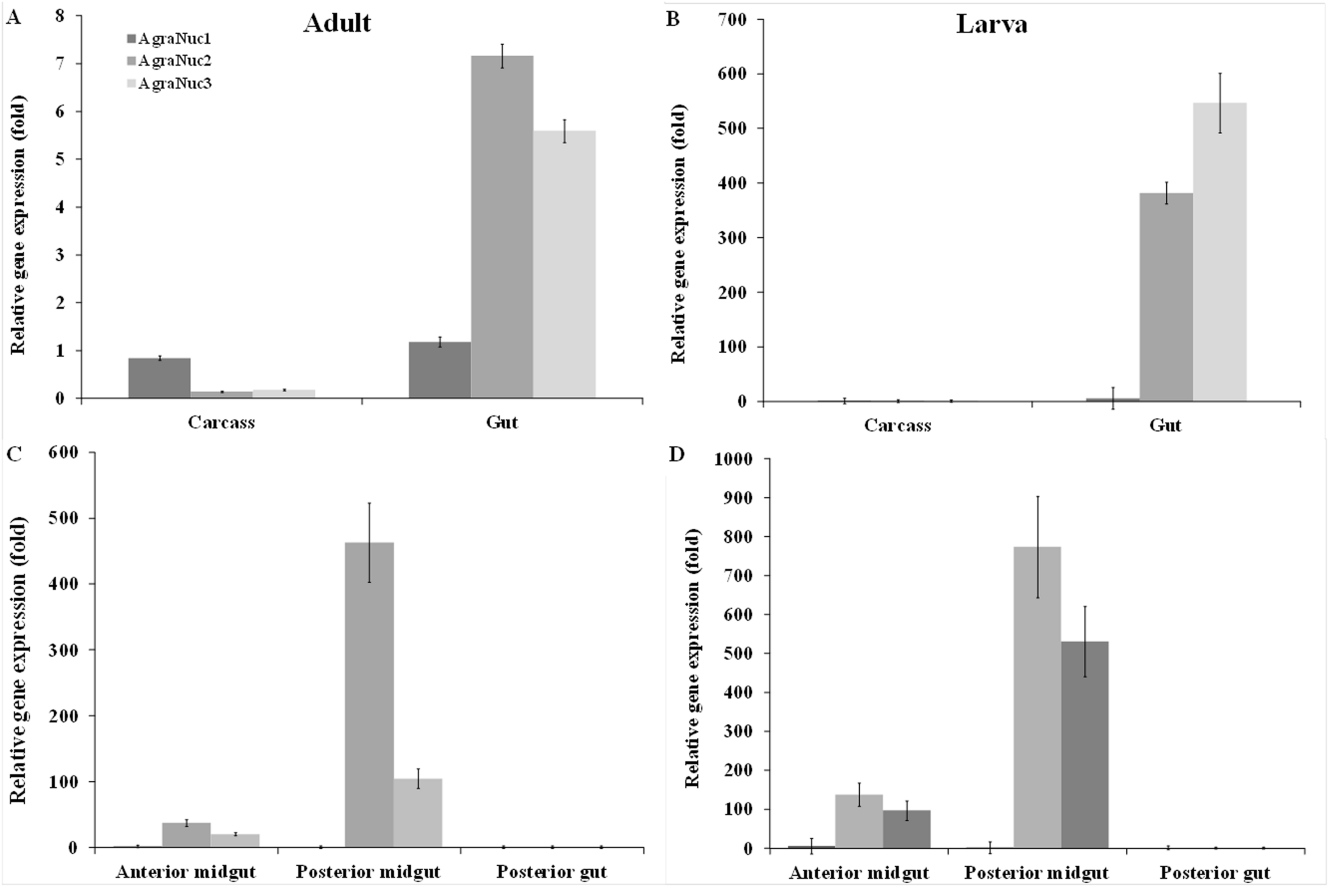
RT-qPCR analysis of CBW nuclease expression at different developmental stages. (A and B) CBW was dissected to obtain the gut and carcass, and nuclease expression was then measured in these samples. The bar chart shows that *AgraNuc1* expression is similar in the gut and carcass of the adult (A) and larvae (B), whereas *AgraNuc2* and *AgraNuc3* are highly expressed in the gut only. (C and D) The insect gut was sectioned into the anterior midgut (AMG), posterior midgut (PMG) and posterior gut (PG), and the expression levels of the nucleases in these sections were evaluated. Higher expression of *AgraNuc2* and *AgraNuc3* was observed in the PMG of both adults (C) and larvae (D), whereas *AgraNuc1* expression was similar in all gut sections. *Agra-β-actin* and *Agra-β-tubulin* were used as reference genes. The relative expression (UA) was calculated based on the lowest expression value that was obtained. Statistical analyses of the average transcripts expression levels were performed using Tukey’s test with a 0.05% significance level for comparisons between treatments.

Digestive enzymes are first produced in gut cells and then secreted in the gut juice [67]. We compared the transcriptional expression profiles of the three above-mentioned CBW nucleases at different developmental stages of the insect, with a focus on the gut. In the first analysis, the carcass and gut from adults and larvae were dissected. The carcass corresponds to the whole insect body without the gut. *AgraNuc1* was found to express at a similar level in the carcass and the gut in both adults and larvae. In contrast, *AgraNuc2* and *AgraNuc3* showed higher expression levels in the gut than in the carcass. The relative expression of *AgraNuc2* and *AgraNuc3* in larvae was 400-600-fold higher than that in adults (Fig 3A and 3B). To examine the expression of *AgraNuc* genes in the gut, we conducted an additional transcriptional analysis of the *AgraNuc* genes, focusing on the anterior midgut (AMG), posterior midgut (PMG) and posterior gut (PG) (S2 Fig). As expected, *AgraNuc1* expression was lower in all gut compartments. However, the PMG displayed significantly higher expression of the *AgraNuc2* and *AgraNuc3* genes in adults (500- and 100-fold) and larvae (800- to 550-fold), respectively (Fig 3C and 3D).

### *AgraNuc* gene silencing resulted in decreased dsRNA digestion by the CBW gut juice

**Fig 4:**
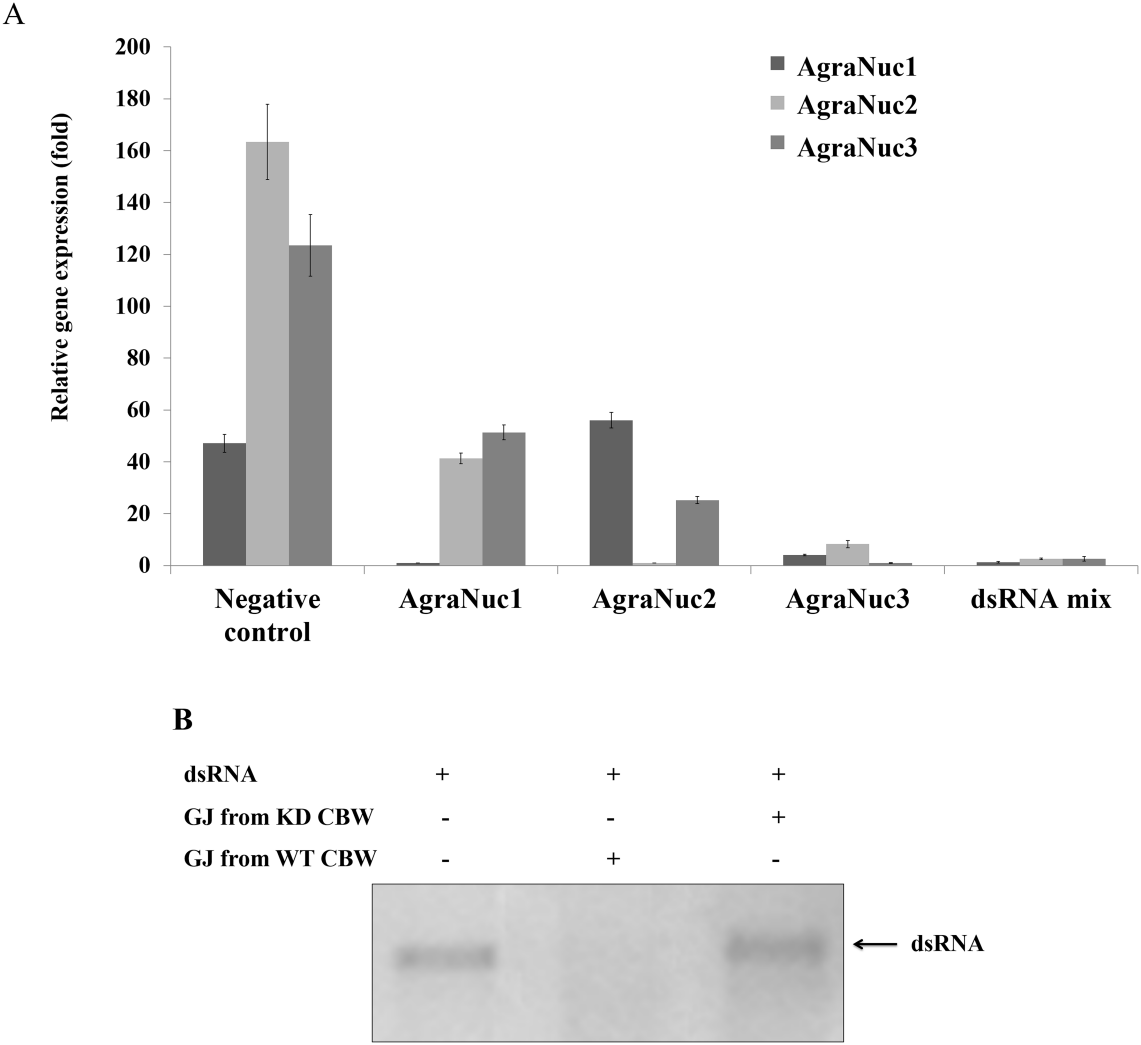
Analysis of CBW nucleases two days after gene silencing by RT-qPCR and dsRNA digestion assay. (A) Insect microinjection was performed with 500 ng of dsRNA against each nuclease and a mixture of all three dsRNAs (in a total of 1500 ng of dsRNA) and the analysis was performed two days after the microinjection. dsRNA against *gus* was used as a negative control, and *Agra-β-actin* and *Agra-β-tubulin* were used as reference genes. The relative expression (UA) was calculated based on the lowest expression value that was obtained. Statistical analyses of the average transcripts expression levels were performed using Tukey’s test with a 0.05% significance level for comparisons between treatments. The bar chart shows that the expression of the nucleases, including each individual nuclease and all three nucleases together, was silenced. (B) dsRNA (~ 200 bp) was incubated with CBW gut juice (GJ) for 30 minutes at 37°C. GJ was collected two days after RNAi nuclease gene silencing, and 1% agarose gel electrophoresis was performed to analyze dsRNA digestion. GJ was collected from uninjected insects and from injected insects with all three nucleases silenced at once. GJ: Gut Juice, KD: knocked down, WT: wild type, CBW: cotton boll weevil.

To determine whether the *AgraNuc* genes contribute to nucleic acid digestion in the gut juice, an RNAi assay of *AgraNuc* gene expression was performed. The downregulation of the *AgraNuc* genes was significant two days after the microinjection of dsRNA into the insect body cavity (Fig 4A) and persisted for seven days after the microinjection (S3 Fig). These results also showed that the microinjection of one specific dsRNA-*AgraNuc* can downregulate the gene expression of another nuclease. For example, dsRNA-*AgraNuc1* also downregulates the gene expression of *AgraNuc2* and *AgraNuc3*. The same phenomenon was found for *AgraNuc2*-dsRNA and *AgraNuc3*-dsRNA (Fig 4A). A dsRNA digestion assay was then performed with gut juice extracted from insects that were given a microinjection of dsRNA against nucleases and from uninjected insects. The gut juice from the uninjected insects resulted in the digestion of dsRNA (lane 3, Fig 4B), whereas the insects that received a microinjection of a mixture of dsRNA against the nucleases showed a decrease in dsRNA digestion (lane 4, Fig 4B). The treatment of insects with dsRNA that targeted *AgraNuc1* and *AgraNuc3* expression lead to dsRNA degradation by the gut juice, suggesting persistent nuclease activity after the dsRNA microinjection. However, a decrease in dsRNA degradation by the gut juice of insects treated with dsRNA-*AgraNuc2* was observed (S4 Fig). This outcome associates the gene silencing of *AgraNuc*2, but not that of *AgraNuc1* and *AgraNuc3*, with a loss of nuclease activity in the gut juice. Due *AgraNuc1* and *AgraNuc3* gene silencing had no effect on dsRNA digestion, it can be affirmed that the *AgraNuc2* knock down is necessary to avoid dsRNA degradation. Furthermore, gene silencing of the three *AgraNuc* genes led to a significant decrease in dsRNA degradation (Fig 4A).

### *AgraNucs* gene silencing improves RNAi efficiency through dsRNA feeding

**Fig 5:**
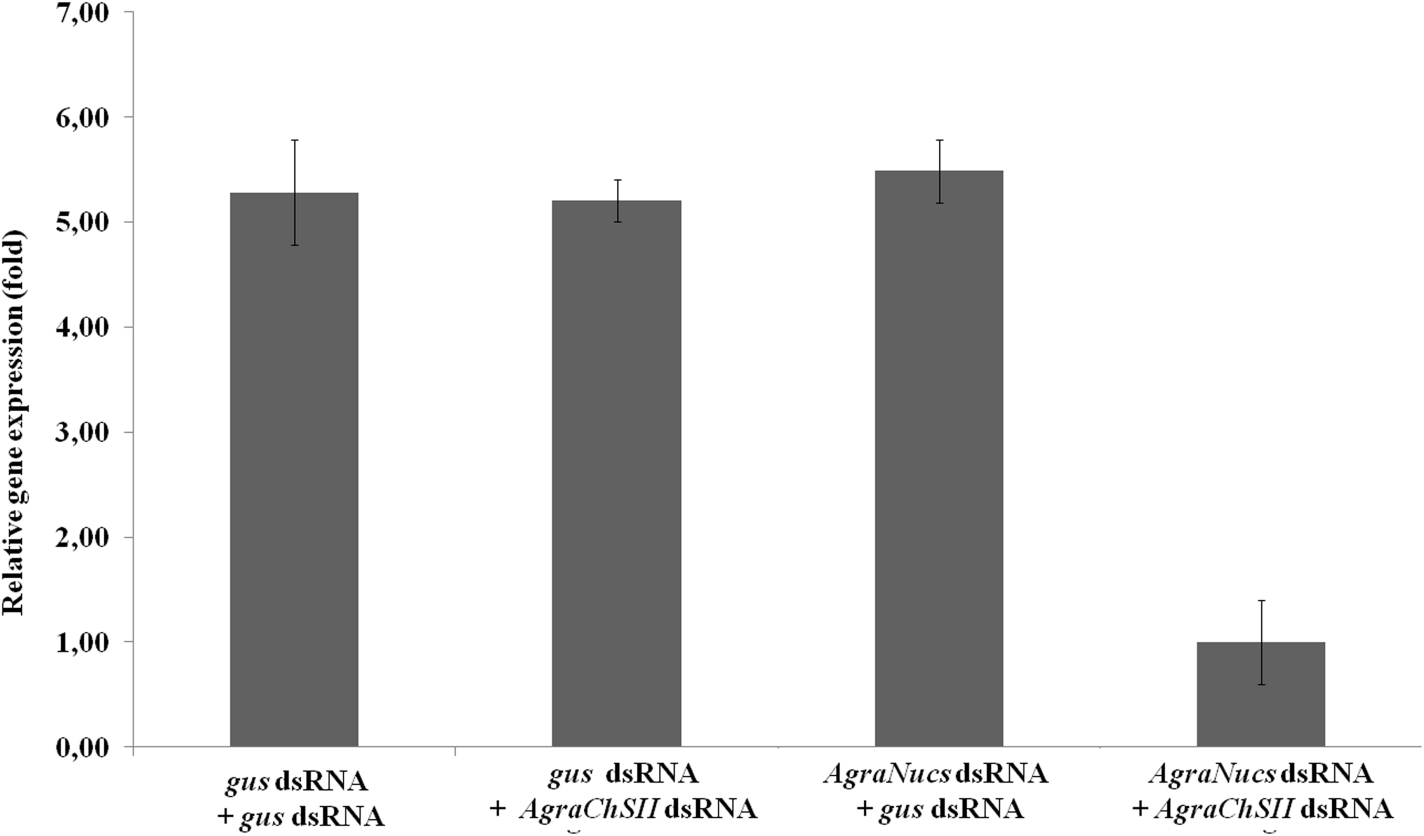
Analysis of CBW *ChSII* gene expression after nuclease gene silencing. Two days after microinjection of the nuclease dsRNA into the CBW body cavity, which silenced the *AgraNuc* genes, the insect was starved for two days, and 500 ng of *AgraChSII* dsRNA was orally administered. The insects with silenced nucleases (fourth bar) showed a decrease in *AgraChSII* transcript expression compared with the control insects (first, second and third bars). RNA extraction, cDNA synthesis and RT-qPCR were performed with the whole insect. dsRNA against *gus* was used as a negative control, and *Agra-β-actin* and *Agra-β-tubulin* were used as reference genes. The relative expression (UA) was calculated based on the lowest expression value that was obtained the average transcripts expression levels were performed using Tukey’s test with a 0.05% significance level for comparisons between treatments.

Since, gene silencing of the three nucleases altogether resulted in decreased dsRNA digestion by the gut juice, dsRNA that targets *chitin synthase II* (*AgraChSII*) was orally administered to CBW adult insects two days after the microinjection of *AgraNucs*-dsRNA and gene silencing verification. Chitin synthase II is responsible for the synthesis of chitin in the peritrophic membrane in the insect midgut [68–70]. The *AgraChSII* gene was silenced by a factor of approximately three after the oral administration of *AgraChSII*-dsRNA to insects in which *AgraNucs* was silenced (Fig 5). Also, insects were counted every 24 hours and mortality was of 85% in insects with nucleases and ChSII silenced, compared to 60% of insects with un-silenced nucleases and silenced ChSII. Control insects injected and fed with *gus* dsRNA had a mortality rate of 15% after 10 days. Oviposition decreased 50% in insects with nucleases and ChSII knock down, in contrast with oviposition of insects injected with *gus* dsRNA and fed with *ChSII* dsRNA. When compared to insects injected and fed with *gus* dsRNA, the oviposition of insects with nucleases and *AgraChSII* knock down was approximately 94% smaller, while the oviposition of insects injected with *gus* dsRNA and fed with *ChSII* dsRNA was approximately 80% smaller (data not shown).

## Discussion

The main advantage of RNA interference technology in controlling crop insect pests is the high specificity for the target gene. Therefore, RNAi is an environmentally safe strategy that decreases off-target effects [19]. Transcriptome analysis can be performed to identify genes that are differentially expressed throughout the lifecycle processes of insects [71]. Using sequence information from the transcriptome, RNAi technology represents an advance in the genetic improvement of crops by conferring resistance to insect pests [72]. However, the delivery of dsRNA to the correct intracellular location and the interaction of dsRNA with the RNAi machinery of the target cell are important issues in the application of this technology. But certain barriers prevent the foreign invading RNA from interfering with the cellular system [73]. As RNA is negatively charged and has a high molecular weight, its movement into the cell is impeded by the lipid bilayer of the cell [74]. In insects, the virus-induced siRNA might saturate the cell to interfere with the artificially delivered dsRNA [75]. The interaction of dsRNA with the cellular receptor and its uptake by the cell also pose important barriers to dsRNA delivery. Nevertheless, the dsRNA concentration and length are critical for the effectiveness of the RNAi response because these features affect the cellular uptake of dsRNA [28, 76–78]. Among others, insect gut nucleases are one of the main barriers to the oral delivery of dsRNA in the application of RNAi as a method for controlling crop insect pests. The oral delivery of dsRNA to its target is hampered by these nucleases because dsRNA catabolism can influence its efficiency. Understanding the interaction between the insects' nuclease activity and dsRNA is fundamental for the advancement of this biotechnological tool, which can be applied in various fields [27, 38, 41, 79–82].

Previous studies demonstrated that dsRNA administration through oral feeding is efficient for gene silencing in a variety of insect species, such as *Helicoverpa armigera* [20, 66, 83], *Diabrotica virgifera* [18], *Aedes aegypti* [84], *Aphis gossypii* [85] and *Apis mellifera* [86]. However, this approach is not efficient in other insects, e.g., *S*. *gregaria* [38], *Acyrthosiphon pisum* [27], *Lygus lineolaris* [87] and *Locusta migratoria* [30]. Coelho and colleagues (2016) showed that the CBW shows a sensitive response to a dsRNA microinjection that interferes in gene silencing. In contrast, Baum and collaborators (2007) demonstrated that the same finding is not observed with the oral administration of dsRNA. This inefficiency is most likely due to gut enzymes that are capable of digesting the nutrients in cotton buds and squares, which are in starch [88] and nucleic acids [89]. It has not been demonstrated, but it can be suggested that CBW evolved to produce gut nucleases because of their feeding habit, being capable to degrade nucleic acids present in the tissues it feeds on. For RNAi technology to perform satisfactorily in crop fields, it is essential to have strategies that protect dsRNA molecules [32, 90–93] from the insect gut, as in CBW.

A study conducted by Shukla and colleagues (2016) [94] demonstrated the degradation of dsRNA in the gut lumen of *Heliothis virescens*. Herein, we report three different nucleases in the gut juice of CBW that are capable of degrading nucleic acid in the insect digestive system. Previously, we identified that the CBW anterior midgut has a pH value of 5.0, whereas the pH of the posterior midgut is 7.0 [26]. In this respect, the dsRNA used in this work was digested by gut nucleases at pH values ranging from 3.5 to 10.0, and at pH ≥ 7.0, the dsRNA digestion was less intense. This data suggests that the nucleases are more active in the anterior midgut of the insect at acidic pH and inactive in the posterior sections with neutral pH. Different insects belonging to various coleopteran families, such as *Hypothenemus hampei* [95], *Dermestes maculatus* [96], *H*. *hampei* [97] and *Sitophilus zeamais* [98], the anterior midgut is acidic, with a pH ranging from 4.5 to 5.0 [99]. Therefore, the pH regulates the nature and activity of the digestive enzymes in the gut lumen [100]. The spatial organization of digestion must be related to the gut compartments; in other words, the action of certain enzymes must be associated with each insect gut lumen section, which present varying pH values [101].

The three nucleases (*AgraNuc1*, *AgraNuc2* and *AgraNuc3*) found in the CBW transcriptome [1] were identified based on the peptide sequence of the *B*. *mori* nuclease [47]. A phylogenetic analysis showed high similarity (>50%) among the CBW nucleases and other insect nucleases. These data, combined with the *in silico* analysis results, suggest that the *AgraNuc* genes encode DNA/RNA non-specific endonucleases and that *AgraNuc2* and *AgraNuc*3 are more likely to be extracellular nucleases than *AgraNuc*1.

The results showed that *AgraNuc2* and *AgraNuc3* are expressed at higher levels in the gut (specifically, in the posterior midgut) in both adults and larvae. These results are in agreement with those reported by Wynant and coworkers (2014) [38], who used the same *B*. *mori* peptide sequence to identify nucleases in the *S*. *gregaria* transcriptome. In that study, nucleases were more expressed in the gut of *S*. *gregaria*. The transcription of *AgraNuc2* and *AgraNuc3* occurs mainly within the PMG, suggesting that these genes contribute to the digestive process. Additionally, this gene expression distribution in the gut supports the compartmentalization of the gut in terms of digestive function [101]. Insects with a peritrophic membrane show endo-ectoperitrophic circulation, which allows the absorption of nutrients that were previously degraded in the endoperitrophic space and enzyme recycling [101]. Hence, the digestive enzymes can be synthesized, secreted in the saliva and posterior midgut, and then transported against the flow to the anterior gut and anterior midgut. This process explains why the transcript expression of the CBW nucleases investigated in this study is higher in the posterior midgut than in the anterior midgut while most of the activity occurs in the acidic anterior midgut. Nevertheless, *AgraNuc1* differs from its two orthologous genes, supporting the notion of a distinct function for this gene. Finally, in the present work, AgraNuc1-3 was found to be probably more associated with nutrition than with cell death.

Nuclease gene silencing was detected two days after microinjection of the dsRNA into the CBW body cavity. Gene silencing by dsRNA against nucleases prevailed even seven days after the microinjection of a single dsRNA or a dsRNA mixture. Interestingly, an identity of a maximum of 11 nucleotides was observed among the nuclease sequences studied in this work. Although it is a short sequence match, it can be enough to cause gene silencing due to the seed region with short matches in the target site [102, 103]. Therefore, the gene silencing of one nuclease by RNAi can interfere with the expression of another nuclease gene by crossed gene silencing. These data suggest that the existence of an RNAi amplification pathway that is independent of RNA-dependent RNA polymerase (RdRp), a pathway that has not been detected in the CBW transcriptome [1]. This pathway is likely unknown but corroborates the findings reported by Wynant and colleagues (2014) [38], who observed the same pattern of crossed gene silencing of nuclease genes in *S*. *gregaria*.

The physiological effects of gene silencing were observed after the incubation of dsRNA with gut juice collected from CBWs that were microinjected with a mixture of dsRNA against the three nucleases. To obtain satisfactory physiological effects with RNAi, it is necessary to sufficiently reduce the expression level of the targeted nucleases and block dsRNA digestion. The physiological effects of nuclease gene silencing can be observed in this study. Silencing of the three CBW nucleases resulted in a reduction of targeted dsRNA digestion upon incubation with the gut juice, showing that the administration of multiple dsRNAs is not always ineffective. Regardless of the competition that might exist among the dsRNAs for the RNAi machinery, the results indicate that the system was not saturated [40, 77, 82]. In contrast, the silencing of the individual nucleases showed decreased dsRNA digestion only in the *AgraNuc2*-silenced insects. It was demonstrated that there was a cross-silencing phenomenon. dsRNA of each nuclease can silence all nucleases but this phenomenon was more intense when ds-*AgraNuc2* was administered. This may be indicative of a threshold of silencing which needs to be crossed, resulting in low levels of nucleases in gut juice in order to see a phenotype. This interesting phenomenon needs to be better studied in the future works. It is noteworthy that the sequences of the three nucleases did not present nucleotide identity higher than 20bp, suggesting the possibility that the decrease in the number of nucleases transcripts may be occurring due to higher levels of gene regulation for these nucleases genes. The data generated in this study corroborate the results reported by Wynant et al. (2014) [38], who identified nucleases that are capable of degrading dsRNA in the *S*. *gregaria* gut and were based on a *B*. *mori* dsRNase identified in the insect gut.

As observed in this study, the presence of nucleases in the gut of crop insect pests is an issue that needs to be addressed because RNAi can be utilized as an effective strategy for pest control. This study demonstrates that the nucleases present in the CBW midgut are capable of being silenced by RNAi, resulting in protection of dsRNA digestion by the gut juice. Thus, further studies are required to develop improved RNAi strategies that can effectively deliver dsRNA to insect pests with protection against insect nucleases, with applicability at field-level [92, 104, 105].

This work contributes to studies on the applicability of the RNAi technique for controlling crop insect pests. The presence of nucleases in the CBW gut leads to a decrease in dsRNA effectiveness. The inhibition of dsRNA digestion improves its uptake by epithelial midgut cells, which is an effect that appears to be mediated by scavenger receptors [106, 107], and becomes significant with a dsRNA greater than 60 bp in length [28]. Digestion of dsRNA can be avoided by the development of approaches that bypass these digestion mechanisms, including the massive production of dsRNA inside chloroplasts [105, 108], dsRNA nanoencapsulation with chitosan [43, 92, 109], and cell-internalizing peptides [91] inside the plant cell. Together, these processes increase dsRNA half-life, enhancing its activity. This field is a novel and vast area associated with plant and crop insect pest biotechnology that requires further study. Thus, overcoming the current barriers to dsRNA delivery would be beneficial for the fruitful application of RNAi to crop fields with the aim of controlling endophytic crop insect pests, such as the cotton boll weevil.

## Supporting information

S1 Fig Conserved amino acids of nucleases. The tree detailed in Fig 1C was constructed from the conserved regions (50 amino acids) of 44 nucleases. Through a Glam2 analysis [55], we verified whether these regions contain the conserved metal binding and catalytic core motifs, which are represented by the Hx_22_Nx_4_Qx_5_Nx_8_E motif and indicated by the black arrows.
(TIF)

S2 Fig Gut sections of CBW. The CBW gut is morphologically divided into four sections: the anterior gut (AG), the anterior midgut (AMG), the posterior midgut (PMG) and the posterior gut (PG).
(TIF)

S3 Fig RT-qPCR analysis of CBW nucleases two and seven days after gene silencing. Insect microinjection was performed with a mixture of nuclease dsRNAs, and the analysis was performed two and seven days after the microinjection. dsRNA against *gus* was used as a negative control, and *Agra-β-actin* and *Agra-β-tubulin* were used as reference genes. The relative expression (UA) was calculated based on the lowest expression value that was obtained. Statistical analyses of the average transcripts expression levels were performed using Tukey’s test with a 0.05% significance level for comparisons between treatments.
(TIF)

S4 Fig dsRNA digestion assay with gut juice from insects with silenced *AgraNuc1*, *AgraNuc2* and *AgraNuc3*. dsRNA (~200 bp) was incubated with CBW gut juice (GJ) that was collected 48 hours after the silencing of nuclease genes by RNAi, and 1% agarose gel electrophoresis was performed to analyze dsRNA digestion. NC: Negative Control; GJ: Gut Juice, KD: knocked down, CBW: cotton boll weevil.
(TIF)
