# Supplementary figures and images for: Nucleases as a barrier to gene silencing in the cotton boll weevil, Anthonomus grandis
Source: PLoS One. 2017 Dec 20;12(12):e0189600. doi: 10.1371/journal.pone.0189600 (PMC5738047; doi:10.1371/journal.pone.0189600)

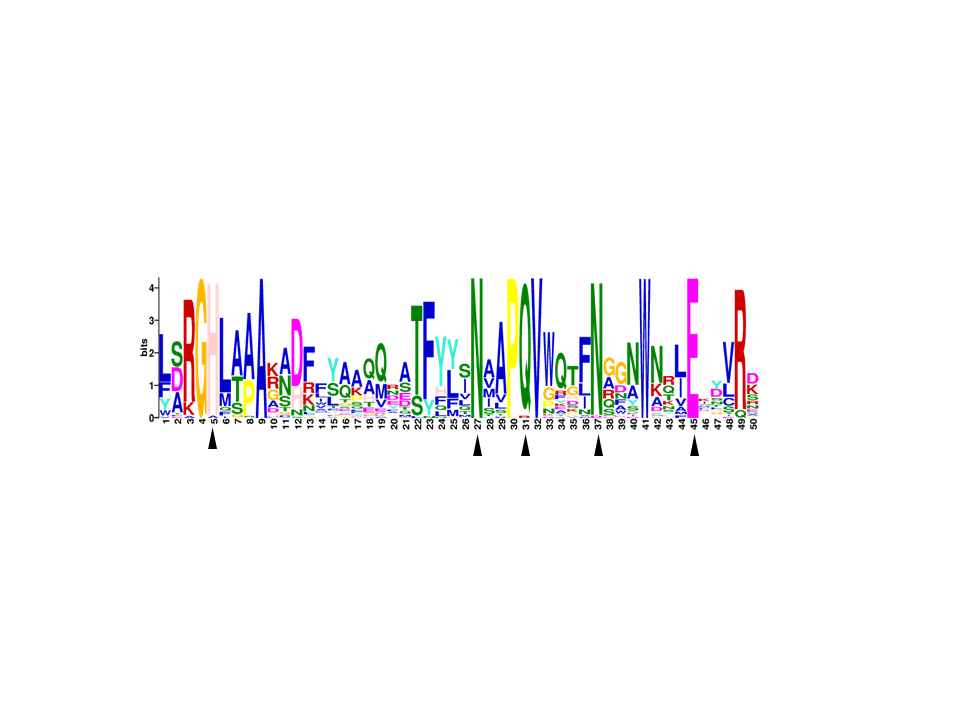

Supplement: S1 Fig — The tree detailed in Fig 1C was constructed from the conserved regions (50 amino acids) of 44 nucleases. Through a Glam2 analysis [55], we verified whether these regions contain the conserved metal binding and catalytic core motifs, which are represented by the Hx22Nx4Qx5Nx8E motif and indicated by the black arrows. (TIF) [file pone.0189600.s001.TIF]

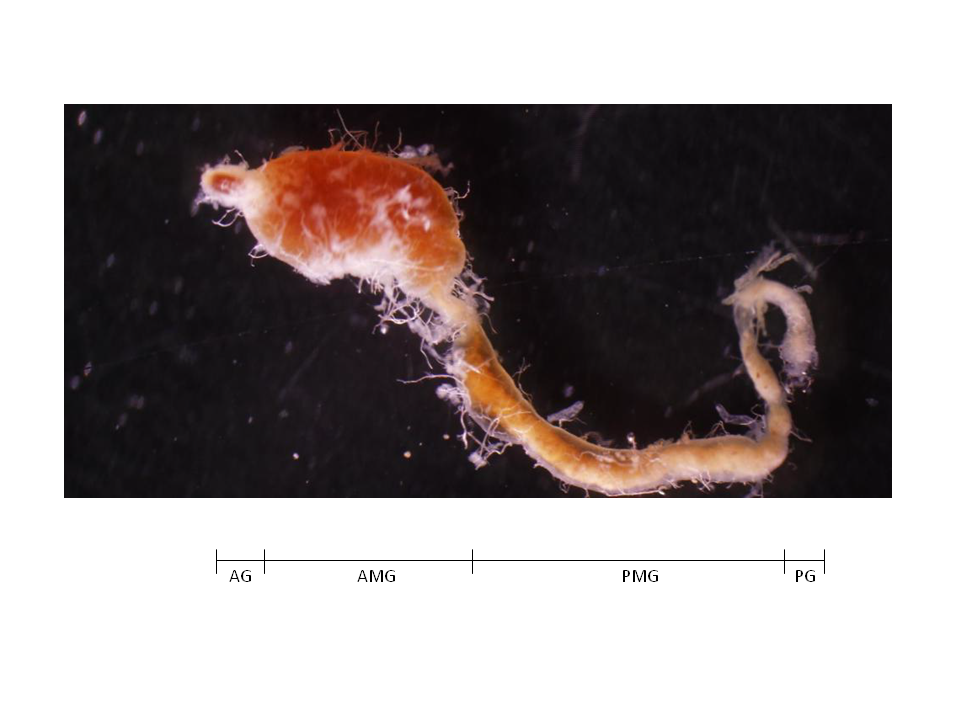

Supplement: S2 Fig — The CBW gut is morphologically divided into four sections: the anterior gut (AG), the anterior midgut (AMG), the posterior midgut (PMG) and the posterior gut (PG). (TIF) [file pone.0189600.s002.TIF]

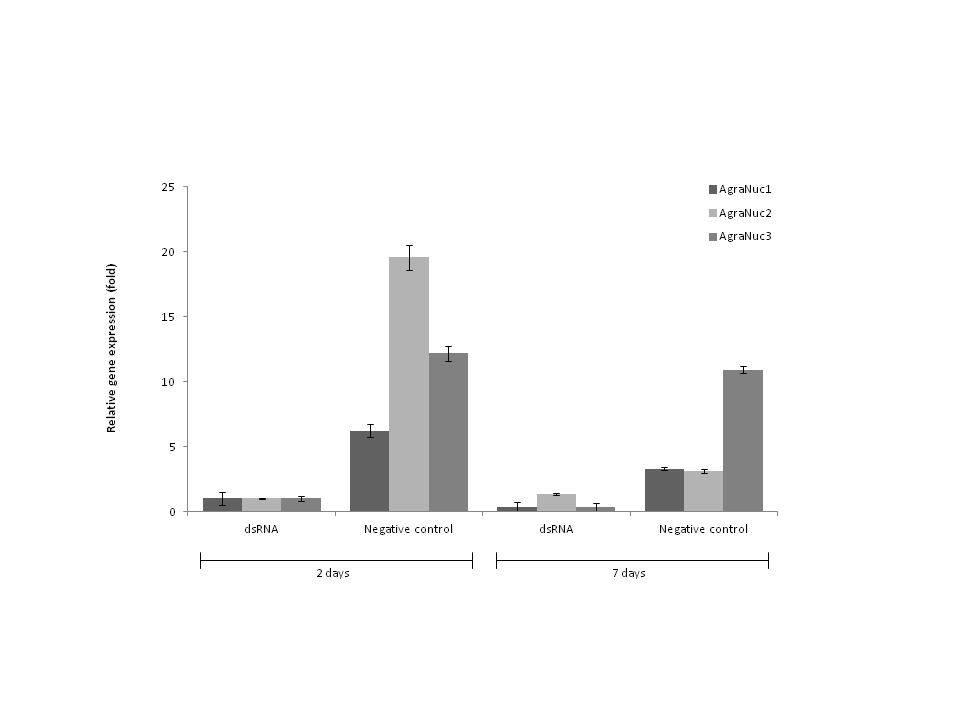

Supplement: S3 Fig — Insect microinjection was performed with a mixture of nuclease dsRNAs, and the analysis was performed two and seven days after the microinjection. dsRNA against gus was used as a negative control, and Agra-β-actin and Agra-β-tubulin were used as reference genes. The relative expression (UA) was calculated based on the lowest expression value that was obtained. Statistical analyses of the average transcripts expression levels were performed using Tukey’s test with a 0.05% significance level for comparisons between treatments. (TIF) [file pone.0189600.s003.TIF]

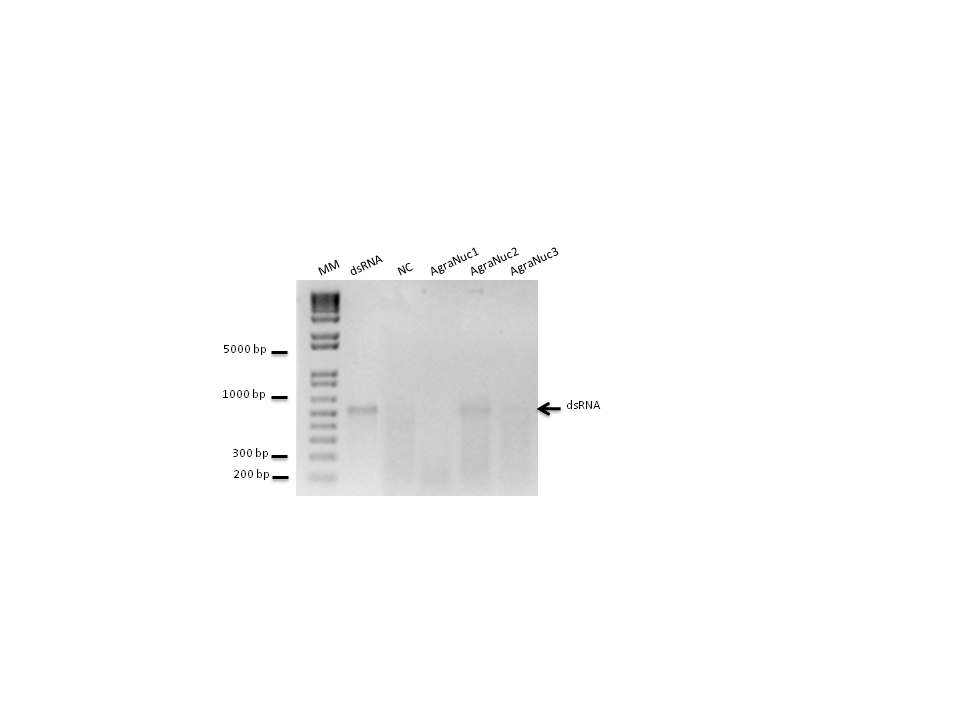

Supplement: S4 Fig — dsRNA (~200 bp) was incubated with CBW gut juice (GJ) that was collected 48 hours after the silencing of nuclease genes by RNAi, and 1% agarose gel electrophoresis was performed to analyze dsRNA digestion. NC: Negative Control; GJ: Gut Juice, KD: knocked down, CBW: cotton boll weevil. (TIF) [file pone.0189600.s004.TIF]
